# Post-transplant predictors of mortality and primary graft dysfunction in lung transplant recipients bridged with veno-venous extracorporeal membrane oxygenation

**DOI:** 10.1007/s10047-026-01584-5

**Published:** 2026-08-19

**Authors:** Nathan T. Kim, Yudai Miyashita, Joshua T. Kim, Taisuke Kaiho, Yuriko Yagi, Chitaru Kurihara

**Affiliations:** 1 Division of Thoracic Surgery, Department of Surgery, Northwestern University Feinberg School of Medicine, 676 N. Saint Clair St., Suite 650, Chicago, IL 60611 USA; 2 Division of Thoracic Surgery, Northwestern University Feinberg School of Medicine, 676 N. Saint Clair St., Suite 650, Chicago, IL 60611 USA

**Keywords:** Veno-venous extracorporeal membrane oxygenation, Lung transplantation, Primary graft dysfunction, Antibody-mediated rejection, Pulmonary embolism.

## Abstract

**Purpose:**

Veno-venous extracorporeal membrane oxygenation (VV-ECMO) is increasingly used to bridge critically ill candidates to lung transplantation, with reported outcomes approaching those of non-ECMO recipients. However, factors associated with early mortality in this high-risk subpopulation remain incompletely defined, particularly when postoperative complications occur after transplantation and may introduce time-dependent bias. We aimed to explore factors associated with 1-year survival among VV-ECMO–bridged lung transplant recipients.

**Methods:**

We performed a single-center retrospective cohort study of adult lung transplant recipients bridged with preoperative VV-ECMO between January 2018 and May 2024. The primary endpoint was 1-year overall survival. Associations with primary graft dysfunction grade 3 were assessed using logistic regression. Because postoperative complications occurred after transplantation, landmark Kaplan–Meier analyses were performed to explore their associations with subsequent survival while reducing immortal time bias.

**Results:**

Forty recipients were included, and 1-year mortality was 20% (8/40). Primary graft dysfunction grade 3 occurred in 16 recipients (40%). Higher pretransplant creatinine and lower albumin showed suggestive associations with primary graft dysfunction grade 3, although these associations did not reach statistical significance in multivariable analysis. In conventional Cox analyses, postoperative pulmonary embolism, dialysis/renal replacement therapy, and antibody-mediated rejection were associated with mortality; however, these findings should be interpreted cautiously because these postoperative complications were time-dependent and the number of deaths was limited. Landmark analyses showed that dialysis/renal replacement therapy by POD30 was associated with worse subsequent survival, whereas the associations for pulmonary embolism by POD90 and antibody-mediated rejection by POD90 were limited by sparse events.

**Conclusions:**

In this single-center cohort of VV-ECMO–bridged lung transplant recipients, postoperative complications including pulmonary embolism, dialysis/renal replacement therapy, and antibody-mediated rejection were associated with mortality in exploratory analyses, with dialysis showing the most consistent association in landmark analysis. Given the small sample size and limited number of events, these findings should be interpreted as hypothesis-generating and require validation in larger multicenter cohorts.

**Supplementary Information:**

The online version contains supplementary material available at https://doi.org/10.1007/s10047-026-01584-5.

## Introduction

Extracorporeal membrane oxygenation (ECMO) has become a common and successful preoperative bridging strategy for critically ill patients awaiting lung transplantation (LTx), extending their ability to survive longer for a lung allograft donation [1–4]. In particular, veno-venous (VV) ECMO allows for reliable pulmonary support and is used more frequently in comparison with veno-arterial (VA) ECMO during the pre-operative period. Pre-transplant risk factors for poor outcomes for lung transplant recipients have been well described and include older age, hepatic or renal dysfunction, and retransplantation [5–7]–in addition to the potential risks that ECMO itself poses to patient vitality (i.e., bleeding, thrombosis, renal injury, etc.) [8, 9]. Nonetheless, several studies agreed that similar outcomes can be achieved in patients bridged with ECMO to those without [4–11]. Despite the growing usage of VV-ECMO as a bridge to transplant, the predictors of mortality in this specific cohort remain incompletely defined–an urgent limitation given the resource-intensive nature of this support system. Moreover, little data is available on post-transplant variables that correlate with increased mortality which may limit clinicians’ ability to optimize postoperative management. To our knowledge, no prior study in VV-ECMO–bridged lung transplant recipients has evaluated risk factors for primary graft dysfunction (PGD) as a distinct outcome, despite PGD being a major cause of early post-transplant mortality. To address these gaps, we conducted a retrospective study of lung transplant recipients bridged with VV-ECMO at a high-volume transplant center. The primary endpoint was 1-year overall survival. Secondary endpoints included PGD grade 3 at 72 h, overall survival over the entire follow-up, and time to Chronic Lung Allograft Dysfunction (CLAD). By shedding light on these distinct but interrelated outcomes, our study aims to inform risk stratification and enhance post-transplant monitoring and management strategies in patients undergoing lung transplantation with VV-ECMO support.

## Materials and methods

### Study design

This study received approval from the Institutional Review Board of Northwestern University (protocol numbers STU00207250 and STU00213616) and patient consent was waived given the retrospective design of the study. Clinical data were gathered retrospectively from electronic medical records and compiled into a secure database maintained at Northwestern University Medical Center in Chicago, Illinois, USA. The study population included adult patients who underwent lung transplantation with preoperative VV-ECMO used as a bridge to transplant between January 2018 and May 2024. Patients undergoing multiorgan transplantation or re-transplantation were excluded. Information collected included patient demographics, existing comorbid conditions, donor-related variables, preoperative laboratory values, intraoperative and postoperative outcomes, and panel reactive antibody levels.

### Definition

#### PGD

PGD was defined in accordance with the 2016 International Society for Heart and Lung Transplantation (ISHLT) consensus-based standardized definition [12], which is classified by ratio of partial pressure of oxygen in arterial blood to fraction of inspired oxygen (Ratio of arterial oxygen partial pressure to fraction of inspired oxygen (PaO2/FiO2)) at 72 h. Grade 1 was defined as a PaO2/FiO2 ratio > 300 mmHg. Grade 2 was defined as a PaO2/FiO2 ratio between 200 and 300 mmHg. Grade 3 was defined as a PaO2/FiO2 ratio < 200 mmHg. Patients on ECMO with chest X-rays displaying pulmonary infiltrations were classified as PGD grade 3.

#### Acute kidney injury (AKI)

AKI was defined using the Risk, Injury, Failure, Loss of kidney function, and End-stage kidney disease (RIFLE) criteria [13]. Risk is defined as a 1.5-fold increase in serum creatinine or a decrease in glomerular filtration rate.

(GFR) by > 25%, or urine output < 0.5 mL/kg/h for 6 h. Injury is defined as a doubling of serum creatinine or a GFR decrease > 50%, or urine output < 0.5 mL/kg/h for 12 h. Failure is defined as a tripling of serum creatinine, a GFR decrease > 75%, or urine output < 0.3 mL/kg/h for 24 h or anuria for 12 h. Loss refers to persistent renal failure requiring renal replacement therapy for > 4 weeks. End-stage kidney disease is defined as complete loss of kidney function for > 3 months. Postoperative dialysis was defined as initiation of any renal replacement therapy after transplantation during the index hospitalization, including intermittent hemodialysis, continuous renal replacement therapy, or peritoneal dialysis (≥ 1 session).

#### Acute cellular rejection (ACR)

ACR is defined by the ISHLT working formulation as the presence of perivascular and interstitial mononuclear inflammatory infiltrates on transbronchial lung biopsy. Rejection is graded on a scale from A0 (no rejection) to A4 (severe rejection) based on the extent and character of the cellular infiltrate [14].

#### Antibody-mediated rejection (AMR)

AMR in lung transplantation is characterized by the presence of donor-specific antibody (DSA) along with histopathologic evidence of capillaritis and complement deposition in lung tissue, combined with clinical graft dysfunction. Transbronchial biopsies were performed for routine surveillance at 1, 3, 6, 9, and 12 months post-transplant, as well as at any time clinical suspicion for rejection arose. The diagnosis of AMR requires an integrated assessment of serologic, histologic, and clinical findings after excluding other potential causes of graft dysfunction [15].

#### CLAD

CLAD was defined via the 2019 ISHLT consensus statement as a significant and persistent decline in the measured forced expiratory volume in 1 s (FEV1) with a difference from the baseline value of ≥ 20% [16]. The baseline value was produced as the mean of the two best FEV1 values following lung transplantation, which were measured at least 3 weeks apart. This decline in FEV₁ was required to be sustained for at least 3 months to confirm chronic dysfunction from transient causes of lung function impairment. CLAD was further subclassified into bronchiolitis obliterans syndrome (BOS) and restrictive allograft syndrome (RAS). BOS is defined physiologically by a ≥ 20% drop in FEV₁ from baseline, accompanied by indices of obstruction (e.g., reduced FEV₁/ forced vital capacity (FVC) ratio) without radiologic pulmonary opacities. RAS, defined by a ≥ 20% decline in FEV₁ (± FVC) from baseline sustained over ≥ 3 months, a ≥ 10% reduction in total lung capacity (TLC) compared to baseline–as measured by body plethysmography, and persistent infiltrates on chest X-ray or high-resolution computed tomography (HRCT) image.

#### ECMO indication criteria

Before undergoing lung transplantation, all intubated patients were managed by a multidisciplinary care team following the clinical guidelines for mechanical ventilation in adults with Acute Respiratory Distress Syndrome (ARDS) set by the American Thoracic Society [17]. ECMO was considered for patients with refractory hypoxemia, defined by a PaO₂ below 55 mmHg, pulse oximetry oxygen saturation under 88%, or a pH less than 7.2. Patients were treated using lung-protective ventilation strategies, including maintaining plateau pressures below 35 mmHg, implementing neuromuscular blockade, and using prone positioning in alignment with Extracorporeal Life Support Organization (ELSO) recommendations [18]. The decision to cannulate was made by the multidisciplinary team, consisting of thoracic surgeons, pulmonologists, intensivists, and ECMO specialists, over teleconference line. To reduce infection risk, central venous catheters (CVCs) were proactively exchanged every seven days, regardless of clinical signs of infection [19, 20]. These replacements were not wire-guided but instead involved insertion at a new vascular access site or a different location within the same vein. Additionally, weekly blood cultures were routinely obtained for all patients on ECMO to monitor for potential bloodstream infections, in line with institutional protocol [21, 22].

### Statistical analysis

Descriptive statistics were used to summarize recipient, donor, and perioperative characteristics. Categorical variables are presented as frequency and percentage, and continuous variables are presented as mean with standard deviation or median with interquartile range (IQR), as appropriate. Comparisons between 1-year survivors and non-survivors were performed using Fisher’s exact test for categorical variables and the Mann–Whitney U test for continuous variables.

Analyses were structured according to the following outcomes: 1-year mortality, overall survival during the entire available follow-up period, and primary graft dysfunction (PGD) grade 3.

For 1-year mortality, defined as death within 365 days after transplantation, baseline and perioperative variables were compared between recipients who were alive and those who died within 1 year. Conventional Cox proportional hazards models were used to explore associations between candidate variables and 1-year mortality. Because postoperative complications, including pulmonary embolism, postoperative dialysis/renal replacement therapy, and antibody-mediated rejection, occurred after transplantation and were inherently time-dependent, the results of conventional Cox models involving these complications were interpreted cautiously.

To further address potential immortal time bias, clinically prespecified landmark analyses were performed. The cohort was restricted to recipients who were alive at each landmark time point, and exposure status was defined according to whether the event had occurred on or before the landmark day. Landmark time points were selected a priori based on the observed clinical timing of each complication: postoperative dialysis/renal replacement therapy was evaluated at postoperative day 30, whereas pulmonary embolism and antibody-mediated rejection were evaluated at postoperative day 90. Follow-up time was re-indexed from the landmark day to death or censoring, and landmark Kaplan–Meier curves were generated to visualize survival differences from the landmark onward.

For overall survival during the entire available follow-up period, Kaplan–Meier survival curves were constructed and compared using the log-rank test. Univariable Cox proportional hazards models were used to evaluate factors associated with overall survival. Variables with a p-value < 0.05 in univariable analyses were considered for inclusion in multivariable Cox proportional hazards models. Hazard ratios (HRs) and 95% confidence intervals (CIs) were reported. These analyses were considered exploratory secondary analyses.

For PGD grade 3, defined as a binary postoperative outcome, univariable logistic regression was used to evaluate candidate predictors. Variables with clinical relevance or a p-value < 0.05 in univariable analyses were considered for multivariable logistic regression. Odds ratios (ORs) and 95% CIs were reported.

All statistical analyses were performed using R software. A two-sided p-value < 0.05 was considered statistically significant.

## Results

### Comparison of patients who died vs. survived within one year

Of the 40 lung transplant recipients who were veno-venous ECMO bridged to transplant, 8 died within one year (20%). There were no statistically significant differences in demographics such as age, sex, and body mass index, as well as comorbidities (e.g., hypertension, diabetes, CKD), lung allocation score, or waiting list time between survivors and non-survivors (Table 1). In contrast, several post-transplant complications demonstrated differences between groups. As Table 2 shows, patients who survived had lower incidence for pulmonary embolism (PE) (9.4% vs. 50%, *p* = 0.02). Regarding timing, postoperative renal replacement therapy occurred early after transplantation (median 3 days; IQR 2–7), with all cases occurring during the index hospitalization. In contrast, PE tended to occur later (median 37 days; IQR 6.5–47), with fewer than half diagnosed by POD30 (3/7) and most by POD90 (6/7) (Supplemental Table 1). Other complications such as cerebrovascular accident (12.5%), bowel ischemia (12.5%) were observed exclusively in the non-survivor group, though these differences were not statistically significant.

**Table 1:** Characteristics of patients

Variable	Survivors (*n* = 32)	Non-Survivors (*n* = 8)	p value
Recipient factors			
Age, years	53.0 (34.0–55.8)	55.5 (41.0–59.8)	0.49
Female	14 (43.8%)	4 (50.0%)	1.00
BMI, kg/m2	26.6 (22.0–28.7)	26.7 (24.4–26.9)	0.91
BSA, m2	1.9 (1.7–2.0)	2.0 (1.9–2.1)	0.23
Smoking history	6 (18.8%)	3 (37.5%)	0.35
Hypertension	11 (34.4%)	4 (50.0%)	0.44
Diabetes	8 (25.0%)	3 (37.5%)	0.66
CKD	2 (6.2%)	1 (12.5%)	0.50
Bilateral	32 (100.0%)	7 (87.5%)	0.20
Days on waiting list	7.0 (4.0–24.3)	8.5 (4.5–18.8)	0.93
Etiology			0.56
ILD	6 (18.8%)	2 (25.0%)	
ARDS	22 (68.8%)	6 (75.0%)	
Other	4 (12.5%)	0 (0.0%)	
Laboratory			
Hemoglobin, g/dL	8.1 (7.1–8.5)	7.5 (7.2–8.3)	0.60
WBC, 1,000/mm3	9.3 (8.1–13.8)	8.2 (7.1–10.8)	0.26
Platelets, 1,000/mm3	172.0 (123.5–223.5)	135 (114–154.8)	0.10
Sodium, mEq/L	140.5 (139.0–146.0)	140.5 (140–141.3)	0.75
BUN, mg/dL	16.0 (13.0–27.5)	15.5 (13–29.3)	0.89
Creatinine, mg/dL	0.6 (0.4–0.8)	0.6 (0.5–0.9)	0.79
ALT, U/L	18.5 (11.0–32.0)	14.0 (11.0–27.8)	0.58
AST, U/L	23.0 (18.3–32.8)	27.5 (15.3–47.5)	0.97
Albumin, g/dL	3.6 (3.2–4.1)	3.1 (2.6–3.9)	0.15
Total bilirubin, mg/dL	0.8 (0.5–1.2)	0.8 (0.4–1.5)	0.70
INR	1.2 (1.1–1.3)	1.2 (1.1–1.3)	0.62
Donor			
Age, years	34.0 (21.8–47)	41.0 (37.8–43.3)	0.17
Female	15 (46.9%)	2 (25.0%)	0.43
Cause of death			0.34
Anoxia	12 (37.5%)	2 (25.0%)	
Head trauma	13 (40.6%)	3 (37.5%)	
Stroke	4 (12.5%)	3 (37.5%)	
Other	3 (9.4%)	0 (0.0%)	

Continuous data are shown as median (interquartile range) and discrete data are shown as number (%). BMI, body mass index; BSA, body surface area; CKD, chronic kidney disease; ILD; interstitial lung disease; COPD, chronic obstructive pulmonary disease; PAH, pulmonary arterial hypertension; ARDS, Acute Respiratory Distress Syndrome; WBC, white blood cell; BUN, blood urea nitrogen; AST, aspartate aminotransferase; ALT, Alanine aminotransferase; INR, international normalized ratio.

**Table 2:** Intra and post operative outcomes of lung transplant recipients

Variable	Survivors (*n* = 32)	Non-Survivors (*n* = 8)	p value
Intraoperative outcome			
Operative time (hours)	8.7 (7.3–10.0)	9.2 (8.3–9.7)	0.61
Intra-op blood transfusion(unit)			
pRBC	9.5 (6.0–16.0)	10.0 (6.5–10.3)	0.85
FFP	3.5 (2.0–7.0)	2.0 (0.8–7.3)	0.59
Plt	2.5 (1.0–4.0)	2.5 (1.0–3.0)	0.75
Ischemic time (hours)	5.8 (5.2–6.4)	5.8 (5.1–6.1)	0.91
VA-ECMO use	31 (96.9%)	7 (87.5%)	0.36
Postoperative outcomes			
de novo DSA	7 (21.9%)	0 (0.0%)	0.31
PGD			
Any grade	20 (62.5%)	7 (87.5%)	0.24
Grade3	11 (34.4%)	5 (62.5%)	0.23
AKI	23 (71.9%)	6 (75.0%)	1.00
Dialysis	11 (34.4%)	4 (50.0%)	0.44
CVA	0 (0.0%)	1 (12.5%)	0.20
Bowel ischemia	0 (0.0%)	1 (12.5%)	0.20
Digital ischemia	1 (3.1%)	1 (12.5%)	0.36
DVT	20 (62.5%)	6 (75.0%)	0.69
PE	3 (9.4%)	4 (50.0%)	0.02
ICU stay (days)*	20.0 (12.3–25.8)	19.5 (15.0–27.0)	0.67
Post transplant ventilator (days)*	5.0 (2.5–17.0)	3.0 (1.0–17.0)	0.62
Hospital stay (days)	36.5 (23.8–47.0)	33.5 (27–43.5)	0.96
ACR	1 (3.1%)	1 (12.5%)	0.36
AMR	1 (3.1%)	2 (25.0%)	0.10
CLAD	5 (15.6%)	0 (0.0%)	0.56
BOS	3 (9.4%)	0 (0.0%)	1.00
RAS/mixed	2 (6.3%)	0 (0.0%)	1.00

Continuous data are shown as median (interquartile range) and discrete data are shown as number (%). pRBC, packed red blood cells; FFP, fresh frozen plasma; Plt, platelets; VA -ECMO, veno-arterial extracorporeal membrane oxygenation; DSA, donor specific antibody; PGD, primary graft dysfunction; AKI, acute kidney injury; CVA, cerebrovascular attack; DVT, deep vein thrombosis; PE, pulmonary embolism; ICU, intensive care unit. *Unknown cases were excluded

### Predictors of PGD grade 3

Out of the cohort of 40 patients, 27 developed PGD of any grade, while 16 of the 27 developed grade 3. In the univariate logistic regression (Table 3) elevated creatinine (OR 19.85, CI 1.11–354.44, *p* = 0.04) was found to be significantly associated with the incidence of PGD grade 3. On the other hand, albumin (OR 0.33, CI 0.11–0.93, *p* = 0.049) was identified to be protective of PGD grade 3 (Table 3. In the multivariable logistic regression (Table 4), creatinine (OR 23.35, CI 0.98–554.11, *p* = 0.051) and Albumin (OR 0.33, CI 0.10–1.03, *p* = 0.06) did not reach statistical significance.

**Table 3:** Univariate logistic regression analysis as a predictor of PGD grade3

Variable	Univariate			
	Odds ratio	95% CI lower	95% CI upper	p value
Recipient factors				
Age, years	0.97	0.93	1.02	0.26
Female	1.40	0.39	5.10	0.60
BMI, kg/m2	0.90	0.74	1.06	0.23
BSA, m2	0.20	0.01	3.83	0.30
Smoking history	2.27	0.50	10.95	0.29
Hypertension	1.00	0.26	3.69	1.00
Diabetes	1.36	0.32	5.63	0.67
CKD	0.73	0.03	8.34	0.81
Etiology				
ILD	0.88	0.16	4.23	0.87
ARDS	1.50	0.38	6.72	0.57
Laboratory				
Hemoglobin, g/dL	1.11	0.63	1.97	0.71
WBC, 1,000/mm3	1.09	0.94	1.28	0.26
Platelets, 1,000/mm3	1.00	0.99	1.01	0.53
Sodium, mEq/L	0.95	0.81	1.10	0.50
BUN, mg/dL	0.99	0.94	1.05	0.83
Creatinine, mg/dL	19.85	1.33	474.56	0.04
ALT, U/L	1.01	0.98	1.05	0.51
AST, U/L	1.01	0.99	1.04	0.32
Albumin, g/dL	0.33	0.10	0.93	0.049
Total bilirubin, mg/dL	1.57	0.84	3.69	0.21
INR	0.06	0.00	5.72	0.24
Donor				
Age, years	0.98	0.93	1.03	0.41
Female	0.71	0.19	2.56	0.60
Intraoperative outcome				
Operative time (hours)	1.01	0.70	1.45	0.98
Ischemic time (hours)	0.61	0.28	1.02	0.17

DSA, donor specific antibody; BMI, body mass index; BSA, body surface area; CKD, chronic kidney disease; WBC, white blood cell; BUN, blood urea nitrogen; AST, aspartate aminotransferase; ALT, Alanine aminotransferase; INR, international normalized ratio.

**Table 4:** Multivariable logistic regression analysis as a predictor of PGD grade3

Variable	Multivariate			
	Odds ratio	95% CI lower	95% CI upper	p value
Laboratory				
Creatinine, mg/dL	23.35	0.98	554.11	0.051
Albumin, g/dL	0.33	0.10	1.03	0.06

PGD; primary graft dysfunction

### Risk factors for 1-year survival

**Fig. 1:**
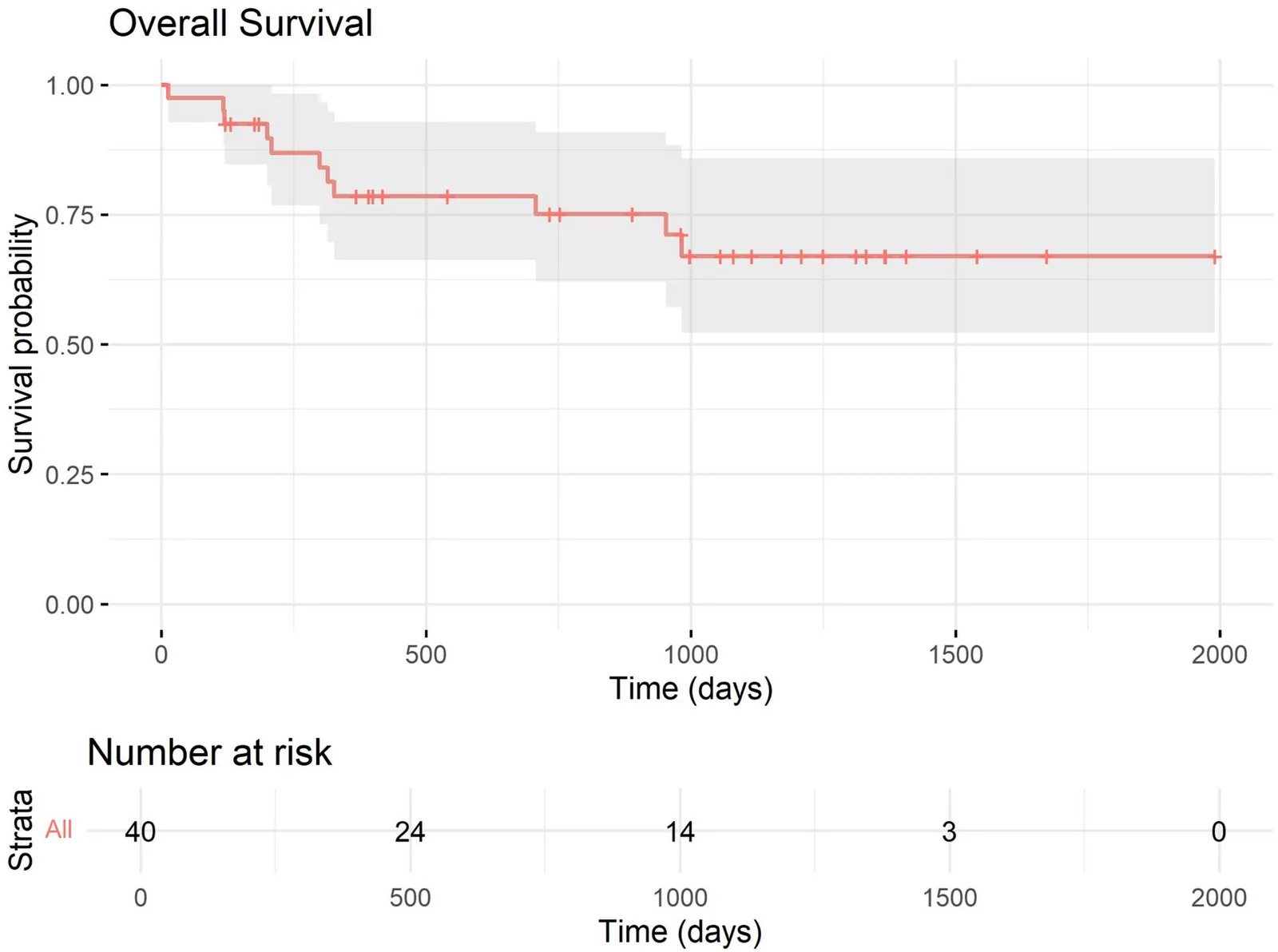
Survival of entire cohort after lung transplantation

**Fig. 2:**
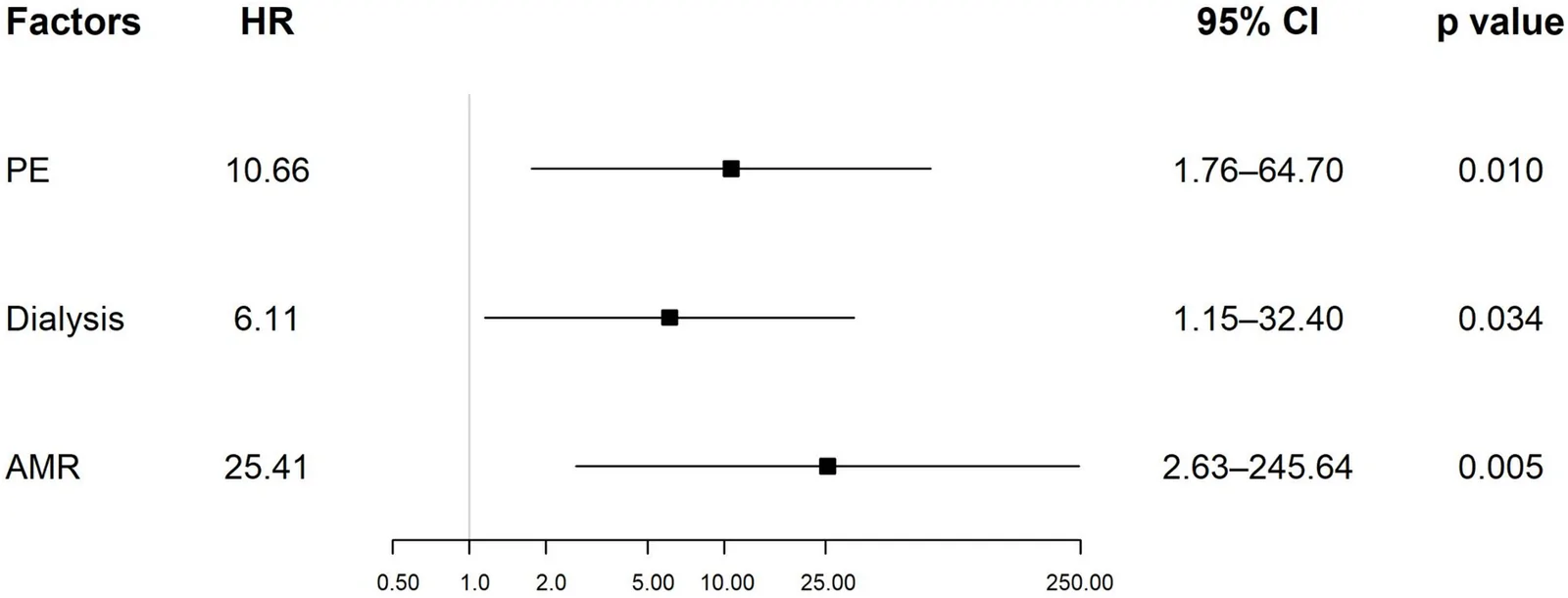
Forest plots showing results of multivariable Cox proportional hazards regression to explore risk factors for 1-year survival. HR; hazard ratio, CI; confidence interval, PE; pulmonary embolism, AMR; Antibody-Mediated Rejection

Out of 40 patients, 29 ultimately survived within the observation period. Figure 1 visualizes a Kaplan-Meier survival curve for the entire cohort, with the steepest drop in survival probability occurring early on, followed by a plateau phase, with no change observed beyond approximately 1000 days. The univariate Cox proportional hazards analyses of the 1-year survival (Table 5) revealed that PE (HR 5.63, 1.40–22.66, *p* = 0.01), postoperative dialysis (HR 7.43, CI 1.50–36.87, *p* = 0.001), and AMR (HR 10.00, CI 1.63–61.40, *p* = 0.01) were significantly associated with increased risk of mortality. In the multivariable model, PE (HR 10.66, CI 1.76–64.70, *p* = 0.01), postoperative dialysis (HR 6.11, CI 1.15–32.40, *p* = 0.03), and AMR (HR 25.41, CI 2.63–245.64, *p* = 0.005) were all found to be associated with mortality (Fig. 2).

**Table 5:** Univariate Cox proportional hazard analysis as a predictor of 1 year survival

Variable	Univariate analysis			
	Hazard Ratio	95% CI lower	95% CI upper	p value
Recipient factors				
Age, years	1.02	0.97	1.07	0.51
Female	1.12	0.28	4.48	0.87
BMI, kg/m2	0.99	0.83	1.18	0.88
BSA, m2	4.11	0.19	87.97	0.37
Smoking history	2.38	0.57	9.98	0.23
Hypertension	1.96	0.49	7.85	0.34
Diabetes	1.54	0.37	6.45	0.56
CKD	1.92	0.24	15.63	0.54
Bilateral	0.21	0.03	1.75	0.15
Etiology				
ILD	1.88	0.38	9.39	0.44
ARDS	1.11	0.22	5.52	0.90
Laboratory				
Hemoglobin, g/dL	0.76	0.36	1.60	0.47
WBC, 1,000/mm3	0.86	0.70	1.07	0.18
Platelets, 1,000/mm3	0.99	0.98	1.00	0.12
Sodium, mEq/L	0.92	0.78	1.09	0.33
BUN, mg/dL	0.99	0.93	1.05	0.71
Creatinine, mg/dL	2.76	0.19	40.13	0.46
ALT, U/L	0.98	0.93	1.03	0.49
AST, U/L	1.00	0.98	1.02	0.84
Albumin, g/dL	0.41	0.14	1.24	0.11
Total bilirubin, mg/dL	0.95	0.47	1.92	0.89
INR	0.12	0.00	19.86	0.41
Donor				
Age, years	1.04	0.98	1.10	0.17
Female	0.49	0.10	2.44	0.39
Intraoperative outcome				
Operative time (hours)	1.05	0.70	1.57	0.82
Ischemic time (hours)	0.86	0.45	1.65	0.65
Postoperative outcomes				
*PGD*				
Any grade	4.15	0.51	33.82	0.18
Grade3	2.52	0.60	10.55	0.21
AKI	1.16	0.23	5.75	0.86
Dialysis	7.43	1.50	36.87	0.01
DVT	1.50	0.30	7.46	0.62
PE	5.63	1.40	22.66	0.01
ACR	2.90	0.35	23.77	0.32
AMR	10.00	1.63	61.40	0.01

CI, confidence interval; BMI, body mass index; BSA, body surface area; CKD, chronic kidney disease; PRA, Panel Reactive Antibody; WBC, white blood cell; BUN, blood urea nitrogen; AST, aspartate aminotransferase; ALT, Alanine aminotransferase; INR, international normalized ratio; DSA, donor specific antibody; PGD, primary graft dysfunction; AKI, acute kidney injury; DVT, deep vein thrombosis; PE, pulmonary embolism; ACR, Acute Cellular Rejection; AMR, Antibody-Mediated Rejection.

### Predictors of CLAD and long term survival

**Fig. 3:**
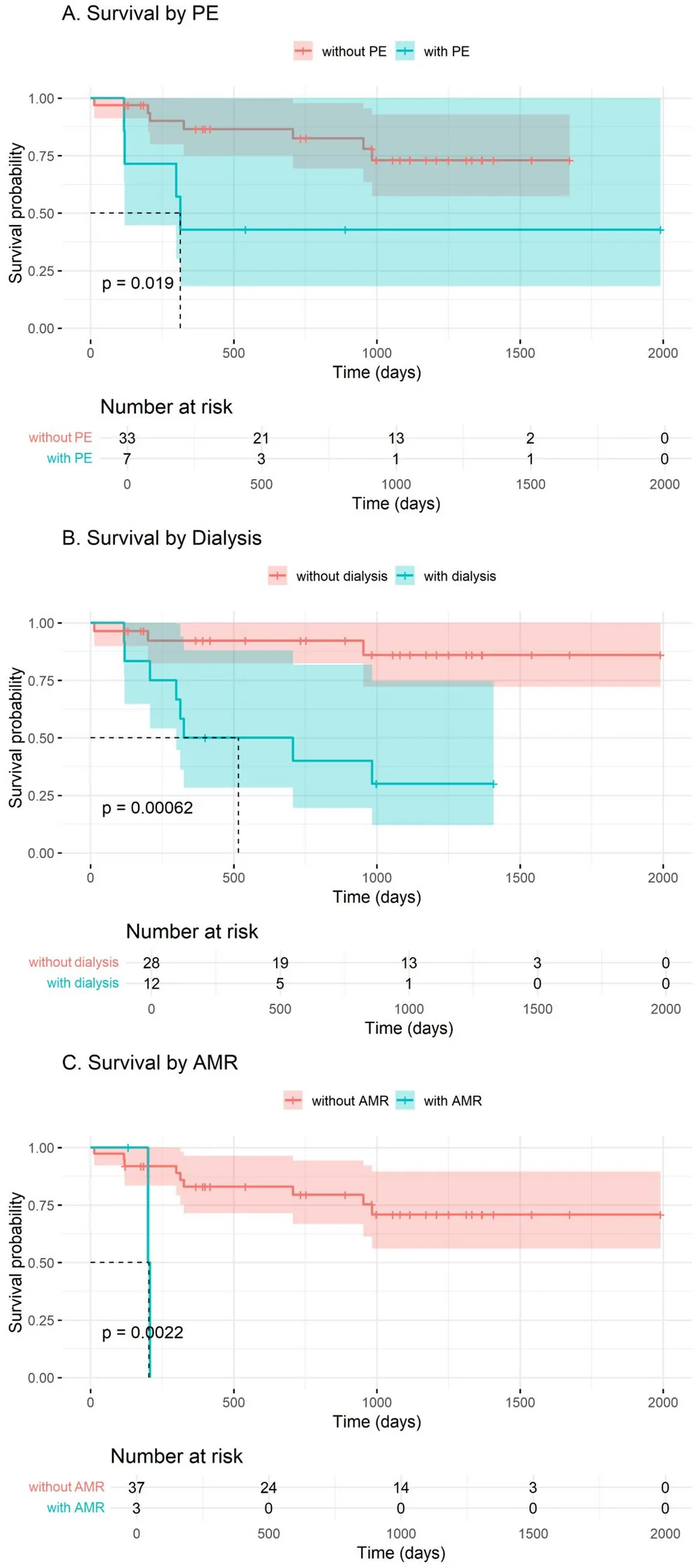
Survival of patients who develop PE, use hemodialysis after lung transplantation, and develop AMR after discharge. PE; pulmonary embolism, AMR; Antibody-Mediated Rejection

Univariate Cox analyses (Supplemental Table 2) did not identify any significant predictors of CLAD. Recipient demographics, comorbidities, donor and intraoperative variables, and early postoperative complications were not associated with CLAD. Consistent with the 1-year survival analysis, PE, postoperative dialysis, and AMR were significant risk factors for long-term mortality (Supplemental Table 3). In the multivariable model (Supplemental Table 4), all three remained independently associated with long term survival: PE (HR 7.14, *p* = 0.01), postoperative dialysis (HR 7.37, *p* = 0.005), and AMR (HR 18.84, *p* = 0.006). Kaplan-Meier curves stratified by PE, dialysis, and AMR (Fig. 3) were generated to display comparisons in survival probabilities in patients experiencing these complications to those without.

**Fig. 4:**
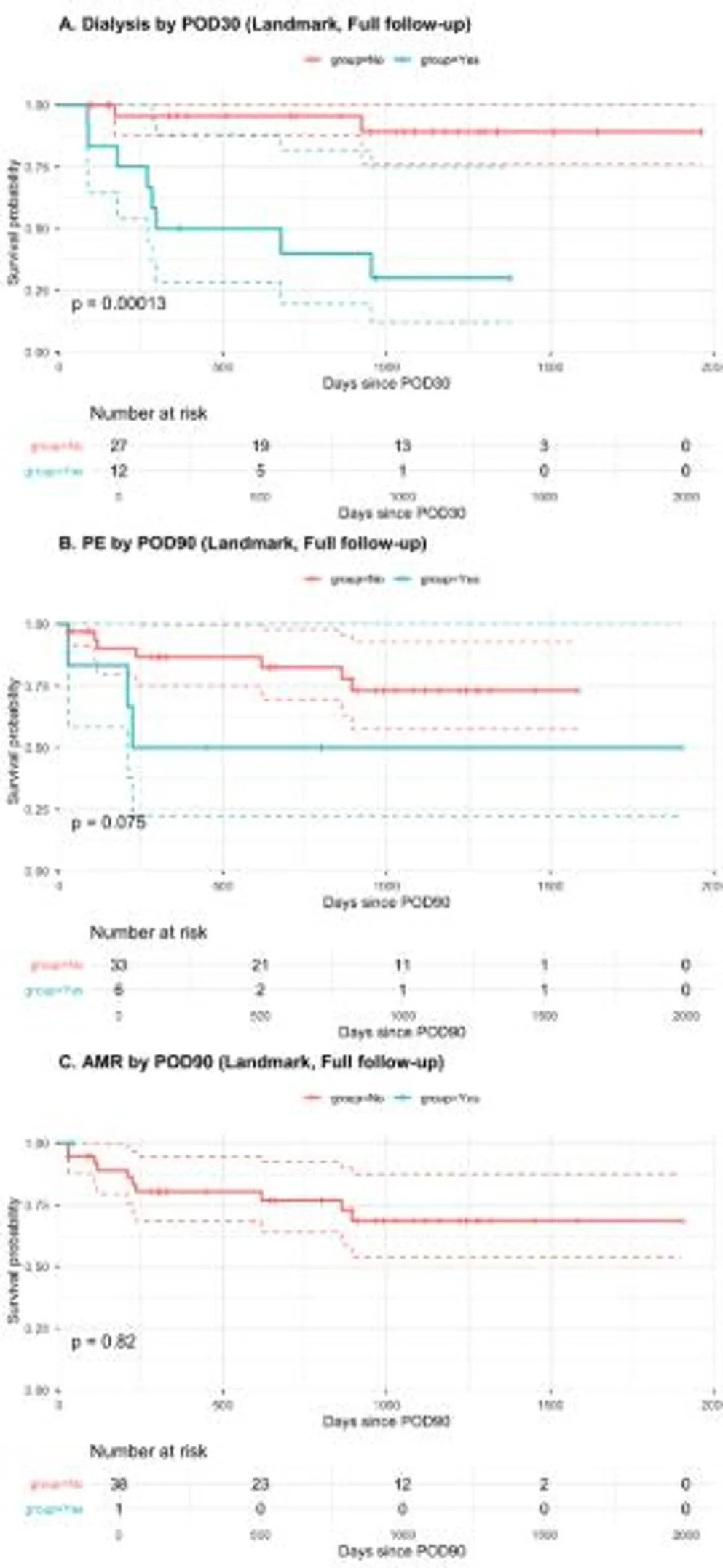
Landmark Kaplan–Meier curves for 1-year overall survival. The cohort was restricted to recipients alive at the landmark day (POD30 or POD90). Exposure status was defined by whether the event occurred on or before the landmark day, and follow-up was re-indexed from the landmark day to death or censoring

To further visualize these associations while accounting for the time-dependent nature of postoperative complications, we generated landmark Kaplan–Meier curves using clinically justified time points and full follow-up (Fig. 4). Specifically, recipients were conditioned on survival to the landmark time point, and groups were defined according to whether each complication occurred on or before the landmark day. Postoperative dialysis was evaluated at POD30, reflecting its early onset during the index hospitalization, whereas PE and AMR were evaluated at POD90 given their later occurrence relative to transplantation. Follow-up was then measured from the landmark day to death or censoring. In the landmark analyses, dialysis/RRT by POD30 was associated with worse subsequent survival. In contrast, PE by POD90 was not statistically significant, and AMR by POD90 was limited by the very small number of exposed patients and events.

## Discussion

Our study aimed to identify post-transplant factors associated with 1-year overall survival in lung transplant recipients bridged with VV-ECMO. Among post-transplant complications, PE, postoperative dialysis/renal replacement therapy, and AMR were associated with 1-year overall mortality in our cohort. Given the time-dependent nature of these events and the limited sample size, these findings should be interpreted as exploratory associations rather than definitive independent prognostic effects.

PE was associated with mortality in conventional Cox analyses; however, this association was not statistically significant in the POD90 landmark analysis. Therefore, the PE-related findings should be interpreted cautiously, and the apparent association in conventional models may have been influenced by the time-dependent nature of PE occurrence and the limited number of events. Nevertheless, thrombotic complications remain clinically important in ECMO-supported patients, particularly in those who are sedated and immobilized. Prior studies have reported an association between awake ECMO strategies and lower mortality rates, potentially mediated by improved mobility [23, 24].

Postoperative dialysis/renal replacement therapy showed the most consistent association with mortality among the postoperative complications evaluated. In conventional Cox analyses, dialysis/RRT was associated with increased mortality, and in the POD30 landmark analysis, dialysis/RRT by POD30 was also associated with worse subsequent survival. This finding supports the clinical relevance of early postoperative renal failure in this high-risk cohort and is consistent with registry studies reporting an association between post-transplant dialysis and worse survival in ECMO-bridged recipients [25]. More broadly, compromised renal function—whether pre-existing or acquired postoperatively—has also been implicated in worse long-term survival across large transplant cohorts [6, 7, 26], demonstrating the importance of perioperative renal protection in shaping long-term outcomes. Post-discharge AMR showed a strong association with mortality in conventional Cox models, but landmark analyses were not estimable due to the very small number of AMR events occurring by POD30/POD90. Accordingly, AMR-related findings should be interpreted cautiously. While the role of AMR in chronic lung allograft dysfunction and late graft loss is well recognized [27], few studies have focused on its impact specifically within ECMO-bridged populations. For example, it has been reported that sensitized lung transplant recipients managed with a perioperative desensitization protocol including eculizumab experienced higher rates of AMR compared to non-sensitized recipients, yet overall survival remained comparable between groups [28]. Taken together, prior literature supports the clinical importance of AMR, and our findings suggest a possible association with mortality in this cohort; however, estimates were imprecise and should be interpreted cautiously.

Regarding pre-transplant factors, prior literature highlighted a variety of predictors such as age, hepatic dysfunction (indicated by high bilirubin levels), and pulmonary hypertension have been reported to increase mortality risk in ECMO-bridged populations [1–30], while one study found traditional concerns such as high body mass index (BMI) to not be a contraindication to ECMO [31]. However, our sample was underpowered to detect such effects definitively, but these remain important considerations in patient selection, as emphasized by international consensus documents [5]. Moreover, several studies point to longer pre-transplant ECMO duration, non-ambulatory status, and retransplantion as strong predictors of mortality in ECMO-bridged patients [1, 8, 23, 24, 32, 33].

Another focal point of our study was identifying predictors of PGD—an early ischemia–reperfusion–driven lung injury distinct from ACR or AMR—that contributes to early morbidity and is associated with subsequent development of CLAD, the predominant cause of poor long-term survival after lung transplantation [34]. Curiously, a retrospective single-center study by Zhao et al. (*n* = 86; 32 received ECMO [5 bridge VV-ECMO, 21 prophylactic intra-/post-op, 6 rescue]) reported that while overall ECMO vs. no ECMO showed similar rates of PGD, patients who got prophylactic ECMO had a lower rate of PGD [35]. Nevertheless, further research is warranted to investigate the link between ECMO and its ability to directly mitigate PGD risk.

In our cohort, 16 out of 40 patients (40%) developed PGD grade 3. On univariate logistic regression, elevated creatinine was associated with increased PGD risk, whereas higher albumin appeared protective. In the multivariable model, both creatinine and albumin showed trends in the expected directions, but neither reached statistical significance. These observations highlight the potential value of renal preservation and nutritional optimization, meriting prospective evaluation in larger cohorts.

This single-center retrospective study limits generalizability to programs with different case mix and protocols. The cohort was small (*n* = 40) with few events, yielding wide confidence intervals and restricting the number of covariates that can be modeled without overfitting; results should therefore be interpreted as exploratory. Data abstracted from the electronic health record are subject to missingness and misclassification, and several clinically relevant variables were unavailable (e.g., pre-transplant ECMO duration, ambulatory status, anticoagulation intensity, and transfusion volume). Although postoperative complications are inherently time-dependent, conventional Cox analyses for 1-year mortality were interpreted cautiously. To reduce potential immortal time bias, we performed clinically prespecified landmark analyses for subsequent survival. In addition, time-dependent Cox models were performed only as exploratory secondary analyses for overall survival during the entire available follow-up period. Nevertheless, residual confounding and sparse-event bias remain important limitations, and effect estimates should be interpreted as exploratory. Nevertheless, residual confounding and sparse-event bias remain important limitations, and effect estimates should be interpreted as exploratory. For CLAD, death was not modeled as a competing risk. We used complete-case analyses without multiple imputation and did not adjust for multiple comparisons, and proportional-hazards assumptions had limited power to assess formally. External validation was not performed.

## Conclusion

In this single-center cohort of VV-ECMO–bridged lung transplant recipients, PE, postoperative dialysis/renal replacement therapy, and AMR were associated with 1-year overall survival, underscoring that post-transplant complications may contribute importantly to outcomes in this high-risk population. As a secondary analysis, for PGD grade 3, creatinine and albumin were associated in univariate analyses, but no independent predictors were confirmed in multivariable models. Larger multicenter studies with time-dependent modeling and sufficient event counts are needed to validate these exploratory associations and to inform the development and evaluation of strategies to improve survival.

## Supplementary Information

Below is the link to the electronic supplementary material.

Supplementary Material 1

## Data Availability

The datasets generated and/or analyzed during the current study are available from the corresponding author on reasonable request.
