# Supplementary material for: Post-transplant predictors of mortality and primary graft dysfunction in lung transplant recipients bridged with veno-venous extracorporeal membrane oxygenation
Source: J Artif Organs. 2026 Aug 19;29(3):57. doi: 10.1007/s10047-026-01584-5 (PMC13490237; doi:10.1007/s10047-026-01584-5)
Supplement: Supplementary file 1 [file 10047_2026_1584_MOESM1_ESM.docx]

Supplementary information for

**Post-Transplant Predictors of Mortality and Primary Graft Dysfunction in Lung Transplant Recipients Bridged with Veno-Venous Extracorporeal Membrane Oxygenation**

Nathan T Kim, Yudai Miyashita, Joshua T Kim, Taisuke Kaiho, Yuriko Yagi, Chitaru Kurihara

**Corresponding Author:**

Chitaru Kurihara, MD

E-mail; [chitaru.kurihara@northwestern.edu](mailto:chitaru.kurihara@northwestern.edu)

This PDF file includes:

Supplemental Tables S1-5

Supplemental Figure 1

| Supplemental Table1. Timing of postoperative complications after lung transplantation and capture within landmark windows | | | | |  |
| --- | --- | --- | --- | --- | --- |
| Event | Total events, n | Median days after LTx (IQR) | By POD30, n/N (%) | By POD90, n/N (%) |  |
| PE | 7 | 37 (6.5-47) | 3/7 (42.9) | 6/7 (85.7) |  |
| DVT | 26 | 7 (1-17) | 22/26 (84.6) | 25/26 (96.2) |  |
| AMR | 3 | 151 (76-160.5) | 0/3 (0.0) | 1/3 (33.3) |  |
| ACR | 2 | 310 (238-382) | 0/2 (0.0) | 0/2 (0.0) |  |
| Post-op dialysis/RRT | 12 | 3 (2-7) | 12/12 (100) | 12/12 (100) |  |
| LTx indicates lung transplantation; POD, postoperative day; IQR, interquartile range; RRT, renal replacement therapy. Days after LTx were calculated from the transplant date (day 0) to the first occurrence/diagnosis date of each complication. “By POD30/By POD90” indicates events occurring on or before POD30/POD90 among patients who experienced the event. | | | | |  |
|  |  |  |  |  |  |
|  |  |  |  |  |  |
|  |  |  |  |  |  |
|  |  |  |  |  |  |

| Supplemental Table 2. Univariate cox proportional hazard analysis as a predictor of CLAD | | | | |  |
| --- | --- | --- | --- | --- | --- |
| Variable | Univariate analysis | | | |  |
|  | Hazard Ratio | 95% CI lower | 95% CI upper | p value |  |
| Recipient factors |  |  |  |  |  |
| Age, years | 0.96 | 0.90 | 1.04 | 0.31 |  |
| Female | 0.72 | 0.12 | 4.37 | 0.72 |  |
| BMI, kg/m2 | 1.11 | 0.93 | 1.33 | 0.23 |  |
| BSA, m2 | 5.08 | 0.09 | 281.19 | 0.43 |  |
| Smoking history | 0.00 | 0.00 | - | 1.00 |  |
| Hypertension | 1.43 | 0.24 | 8.56 | 0.70 |  |
| Diabetes | 1.54 | 0.26 | 9.23 | 0.64 |  |
| CKD | 3584937079.57 | 0.00 | - | 1.00 |  |
| Bilateral | 9377000.83 | 0.00 | - | 1.00 |  |
| PRA | 0.91 | 0.15 | 5.50 | 0.92 |  |
| Etiology |  |  |  |  |  |
| ILD | 0.00 | 0.00 | Inf | 1.00 |  |
| ARDS | 1.58 | 0.17 | 14.68 | 0.69 |  |
| Laboratory |  |  |  |  |  |
| Hemoglobin, g/dL | 1.89 | 0.83 | 4.29 | 0.13 |  |
| WBC, 1,000/mm3 | 0.95 | 0.77 | 1.19 | 0.68 |  |
| Platelets, 1,000/mm3 | 1.00 | 0.98 | 1.01 | 0.46 |  |
| Sodium, mEq/L | 1.08 | 0.88 | 1.31 | 0.46 |  |
| BUN, mg/dL | 1.03 | 0.97 | 1.09 | 0.32 |  |
| Creatinine, mg/dL | 0.01 | 0.00 | 9.23 | 0.19 |  |
| ALT, U/L | 0.98 | 0.91 | 1.04 | 0.49 |  |
| AST, U/L | 1.00 | 0.97 | 1.03 | 0.80 |  |
| Albumin, g/dL | 2.97 | 0.62 | 14.13 | 0.17 |  |
| Total bilirubin, mg/dL | 1.54 | 0.89 | 2.66 | 0.12 |  |
| INR | 0.01 | 0.00 | 23.95 | 0.24 |  |
| Donor |  |  |  |  |  |
| Age, years | 1.02 | 0.96 | 1.08 | 0.54 |  |
| Female | 0.37 | 0.04 | 3.38 | 0.38 |  |
| Intraoperative outcome |  |  |  |  |  |
| Operative time (hours) | 1.07 | 0.58 | 1.98 | 0.83 |  |
| Ischemic time (hours) | 2.15 | 0.92 | 5.04 | 0.08 |  |
| Postoperative outcomes |  |  |  |  |  |
| PGD |  |  |  |  |  |
| Any grade | 0.92 | 0.15 | 5.58 | 0.93 |  |
| Grade3 | 0.30 | 0.03 | 2.76 | 0.29 |  |
| AKI | 0.54 | 0.09 | 3.24 | 0.50 |  |
| Dialysis | 1.10 | 0.12 | 10.05 | 0.93 |  |
| DVT | 0.34 | 0.06 | 2.04 | 0.24 |  |
| PE | 0.00 | 0.00 | - | 1.00 |  |
| ACR | 0.00 | 0.00 | - | 1.00 |  |
| AMR | NE | NE | NE | NE |  |
| BMI, body mass index; BSA, body surface area; CKD, chronic kidney disease; PRA, Panel Reactive Antibody; WBC, white blood cell; BUN, blood urea nitrogen; AST, aspartate aminotransferase; ALT, Alanine aminotransferase; INR, international normalized ratio; DSA, donor specific antibody; PGD, primary graft dysfunction; AKI, acute kidney injury; DVT, deep vein thrombosis; PE, pulmonary embolism; ACR, Acute Cellular Rejection; AMR, Antibody-Mediated Rejection. *NE: not estimable. | | | | |  |
|  |  |  |  |  |  |
|  |  |  |  |  |  |
|  |  |  |  |  |  |
|  |  |  |  |  |  |

| Supplemental Table 3. Univariate cox proportional hazard analysis as a predictor of long term survival | | | | |  |
| --- | --- | --- | --- | --- | --- |
| Variable | Univariate analysis | | | |  |
|  | Hazard Ratio | 95% CI lower | 95% CI upper | p value |  |
| Recipient factors |  |  |  |  |  |
| Age, years | 1.02 | 0.97 | 1.07 | 0.45 |  |
| Female | 1.48 | 0.45 | 4.88 | 0.52 |  |
| BMI, kg/m2 | 1.04 | 0.91 | 1.20 | 0.55 |  |
| BSA, m2 | 2.44 | 0.18 | 33.03 | 0.50 |  |
| Smoking history | 1.99 | 0.51 | 7.84 | 0.33 |  |
| Hypertension | 1.62 | 0.49 | 5.33 | 0.42 |  |
| Diabetes | 1.49 | 0.44 | 5.11 | 0.52 |  |
| CKD | 1.92 | 0.24 | 15.63 | 0.54 |  |
| Bilateral | 0.21 | 0.03 | 1.75 | 0.15 |  |
| PRA | 0.79 | 0.24 | 2.58 | 0.69 |  |
| Etiology |  |  |  |  |  |
| ILD | 1.39 | 0.30 | 6.49 | 0.67 |  |
| ARDS | 1.45 | 0.31 | 6.76 | 0.64 |  |
| Laboratory |  |  |  |  |  |
| Hemoglobin, g/dL | 0.97 | 0.55 | 1.73 | 0.93 |  |
| WBC, 1,000/mm3 | 0.93 | 0.80 | 1.08 | 0.35 |  |
| Platelets, 1,000/mm3 | 0.99 | 0.98 | 1.00 | 0.08 |  |
| Sodium, mEq/L | 0.96 | 0.84 | 1.10 | 0.60 |  |
| BUN, mg/dL | 1.00 | 0.96 | 1.04 | 0.97 |  |
| Creatinine, mg/dL | 2.06 | 0.21 | 20.50 | 0.54 |  |
| ALT, U/L | 1.01 | 0.98 | 1.04 | 0.40 |  |
| AST, U/L | 1.01 | 0.99 | 1.02 | 0.29 |  |
| Albumin, g/dL | 0.53 | 0.21 | 1.32 | 0.17 |  |
| Total bilirubin, mg/dL | 1.26 | 0.86 | 1.84 | 0.24 |  |
| INR | 0.07 | 0.00 | 5.95 | 0.24 |  |
| Donor |  |  |  |  |  |
| Age, years | 1.03 | 0.98 | 1.07 | 0.27 |  |
| Female | 0.89 | 0.26 | 3.06 | 0.86 |  |
| Intraoperative outcome |  |  |  |  |  |
| Operative time (hours) | 1.05 | 0.73 | 1.51 | 0.81 |  |
| Ischemic time (hours) | 0.89 | 0.52 | 1.51 | 0.67 |  |
| Postoperative outcomes |  |  |  |  |  |
| PGD |  |  |  |  |  |
| Any grade | 2.62 | 0.56 | 12.14 | 0.22 |  |
| Grade3 | 2.58 | 0.75 | 8.80 | 0.13 |  |
| AKI | 1.77 | 0.38 | 8.19 | 0.47 |  |
| Dialysis | 7.31 | 1.93 | 27.70 | 0.003 |  |
| DVT | 2.23 | 0.48 | 10.33 | 0.30 |  |
| PE | 4.02 | 1.15 | 14.11 | 0.03 |  |
| ACR | 2.90 | 0.35 | 23.77 | 0.32 |  |
| AMR | 10.00 | 1.63 | 61.40 | 0.01 |  |
| CI, confidence interval; BMI, body mass index; BSA, body surface area; CKD, chronic kidney disease; PRA, Panel Reactive Antibody; WBC, white blood cell; BUN, blood urea nitrogen; AST, aspartate aminotransferase; ALT, Alanine aminotransferase; INR, international normalized ratio; DSA, donor specific antibody; PGD, primary graft dysfunction; AKI, acute kidney injury; DVT, deep vein thrombosis; PE, pulmonary embolism; ACR, Acute Cellular Rejection; AMR, Antibody-Mediated Rejection. | | | | |  |
|  |  |  |  |  |  |
|  |  |  |  |  |  |
|  |  |  |  |  |  |
|  |  |  |  |  |  |

| Supplemental Table 4. Multivariate cox proportional hazard analysis as a predictor of long term survival | | | | |
| --- | --- | --- | --- | --- |
| Variable | Multivariate analysis | | | |
|  | Hazard Ratio | 95% CI lower | 95% CI upper | p value |
| PE | 7.14 | 1.55 | 32.86 | 0.01 |
| Dialysis | 7.37 | 1.82 | 29.82 | 0.005 |
| AMR | 18.84 | 2.34 | 151.53 | 0.006 |
| CI, confidence interval; PE, pulmonary embolism, AMR, Antibody-Mediated Rejection. | | | | |

| Supplemental Table 5. Time-dependent Cox regression for overall survival after lung transplantation | | | | |
| --- | --- | --- | --- | --- |
| Variable | Multivariate analysis | | | |
|  | Hazard Ratio | 95% CI lower | 95% CI upper | p value |
| PE | 28.55 | 2.24 | 363.05 | 0.01 |
| Dialysis | 16.73 | 1.41 | 197.85 | 0.025 |
| AMR | 243.63 | 11.03 | 5379.21 | 0.001 |
| CI, confidence interval; PE, pulmonary embolism, AMR, Antibody-Mediated Rejection. | | | |  |
